# Fluid and Biopsy Based Biomarkers in Parkinson’s Disease

**DOI:** 10.1007/s13311-023-01379-z

**Published:** 2023-05-03

**Authors:** David G. Coughlin, David J. Irwin

**Affiliations:** 1 Department of Neurosciences, University of California San Diego, 9444 Medical Center Drive, ECOB 03-021, MCC 0886, La Jolla, CA 92037 USA; 2 Department of Neurology, University of Pennsylvania, Philadelphia, PA 19104 USA

**Keywords:** Parkinson’s disease, Alpha-synuclein, Real-time quaking induced conversion, Protein misfolding cyclic amplification, Immunofluorescence, Biopsy

## Abstract

Several advances in fluid and tissue-based biomarkers for use in Parkinson’s disease (PD) and other synucleinopathies have been made in the last several years. While work continues on species of alpha-synuclein (aSyn) and other proteins which can be measured from spinal fluid and plasma samples, immunohistochemistry and immunofluorescence from peripheral tissue biopsies and alpha-synuclein seeding amplification assays (aSyn-SAA: including real-time quaking induced conversion (RT-QuIC) and protein misfolding cyclic amplification (PMCA)) now offer a crucial advancement in their ability to identify aSyn species in PD patients in a categorical fashion (i.e., of aSyn + vs aSyn −); to augment clinical diagnosis however, aSyn-specific assays that have quantitative relevance to pathological burden remain an unmet need. Alzheimer’s disease (AD) co-pathology is commonly found postmortem in PD, especially in those who develop dementia, and dementia with Lewy bodies (DLB). Biofluid biomarkers for tau and amyloid beta species can detect AD co-pathology in PD and DLB, which does have relevance for prognosis, but further work is needed to understand the interplay of aSyn tau, amyloid beta, and other pathological changes to generate comprehensive biomarker profiles for patients in a manner translatable to clinical trial design and individualized therapies.

**Supplementary Information:**

The online version contains supplementary material available at 10.1007/s13311-023-01379-z.

## Introduction

Parkinson’s disease (PD) is pathologically characterized by inclusions of alpha-synuclein (aSyn) that compose Lewy bodies and Lewy neurites [1]. These inclusions are found in fairly stereotyped patterns that progress from brainstem nuclei, to limbic regions and lastly to neocortical areas [2]. At this time, a definitive diagnosis of PD can only be rendered after neuropathological assessments are performed, with levels of clinically established and clinically probable certainties being attainable during life [3]. Clinical diagnostic accuracy for PD has varied among studies over the last several decades and ranges from 50% to greater than 90% [4–9]. Factors that tend to relate to lower diagnostic accuracy are an older age at onset and a shorter degree of disease duration at time of assessment or a lower amount of clinical follow-up time [4, 9]. Thus, the diagnostic standard remains postmortem neuropathological diagnosis until a method to reliably detect aSyn in vivo is developed. Most biomarker studies rely on patients who have been clinically diagnosed with PD who do not go on to have autopsy validation. While this creates some uncertainty regarding the accuracy of diagnosis and this may be problematic in developing novel biomarkers, the current clinical criteria for PD are felt to have high specificity [9]. Furthermore, because there is no currently accepted quantitative aSyn biomarker, studies of these candidate biomarkers are compared to clinical metrics like motor severity or cognition which can be influenced by many factors and are fundamentally indirect measures of disease activity. While aSyn-specific biomarkers remain a critical unmet need for the field, they are especially needed for application in early disease when clinical diagnostic accuracy is at its lowest and also when disease-modifying interventions may have the greater utility.

Over the last decade, there has been considerable advancements in fluid and tissue-based assays in PD. Early work focused on CSF aSyn species including total aSyn, phosphorylated aSyn, and oligomeric aSyn species using immunoassays [10–12]. Plasma aSyn assays are under development as well [13, 14]. More recently, aSyn deposits have been noted in a variety of peripheral tissues of PD patients, including skin, submandibular glad, colon, and nasal mucosa and these observations have led to the development of methods to detect these deposits through immunohistochemistry or immunofluorescence methods [15, 16]. Additionally, the observations that pathologically misfolded aSyn species may induce sequential templating of normal monomeric aSyn in a prion-like fashion, has led to the development of aSyn-seeding amplification assays (aSyn-SAAs), which use these properties to identify patients who harbor pathogenic aSyn seeds in spinal fluid and peripheral tissues [17–21]. While some of these assays are still under development in the research setting, others are reaching levels of standardization and interlaboratory variability rapidly approaching possible acceptable levels for clinical use.

aSyn aggregates in Lewy bodies and Lewy neurites are the primary neuropathology and gold-standard for diagnosis of PD and their burden is roughly related to severity of disease and certain disease features like dementia [22–26]. However, multiple biological factors, even sex, can influence phenotypic expression of pathological burden [27, 28]. Additionally, it is exceedingly common in autopsy studies that other co-pathologies aside from aSyn are found; approximately 35–50% of PD patients with dementia with have moderate to high levels of AD neuropathologic change [29–33]. This number is considerably higher in DLB, where rates of moderate or severe AD co-pathology can reach 70% or greater [34, 35]. The presence of the AD co-pathology is well described to be related to older age of onset, faster time to dementia, decreased overall survival, greater likelihood of an akinetic-rigid motor phenotype, and specific cognitive features [31, 32, 36–42]. AD biomarkers are established using a framework for biological classification of AD based on positivity for amyloid-beta, tau, and neurodegeneration (A/T/N) [43]. A similar approach is understudied in PD and related synucleinopathies, but early work suggests that CSF AD biomarkers can be used to detect the presence of these AD co-pathologies in PD and predict cognitive and overall prognostic outcomes which could have utility in clinical care and trial design [44–51]. However, the application of AD CSF biomarkers in clinical care for PD is unclear, as biological factors related to aSyn may influence AD biomarkers in a manner independent from AD pathology, necessitating PD-specific diagnostic cut-points, but further studies with autopsy-confirmation are needed [46, 47, 52]. The presence of AD co-pathology is not universal, nor is it exclusively linked to worse prognosis; there are many cases of “pure” aSyn cases with fulminant presentations and precipitous clinical courses [38, 53, 54]. Finally, neuropathology of aging is complex and often includes additional mixed pathologies in PD and related disorders such as cerebrovascular disease, TDP-43 inclusions, age-related glial tau inclusions (ARTAG) and others which cannot be reliable measured through fluid assays at this time [55–57]. Here, we review the state of the science for aSyn related biomarkers in CSF, plasma, biopsy detection from peripheral tissues, and aSyn seeding amplification assays for PD with a focus on autopsy-confirmation which is a critical to help translate biomarker research into clinical practice.

## CSF aSyn Assays

In vivo CSF aSyn levels have been studied extensively, but due to conflicting results and significant overlap in values with healthy controls and other disease states their current utility in PD is limited until further refinement occurs [11]. There are several technical considerations which make measuring CSF aSyn challenging. First, there is relatively little aSyn that is present in spinal fluid, on the orders ng/ml. There is high amounts in peripheral blood in red blood cells which can easily contaminate specimens [58]. In many studies, samples have had to be discarded if there are significant degrees of hemoglobin contamination in CSF samples [59, 60]. Polypropylene collection tubes are recommended for use in some assays to prevent loss of CSF aSyn, and there is variability even amongst different tube vendors in their effects on aSyn levels [61]. Time to storage, number of freeze–thaw cycles and other pre-analytic variables affects measured CSF aSyn levels [61, 62]. Most of these studies have used ELISA assays or the bead-based Luminex xMap platform which are calibrated against measurements made with recombinant aSyn (Table 1). ELISA assays may use different aSyn antibodies for capture and detection which may account for some degree of variability observed. For ELISA protocols, there tends to be relatively high intra-assay precision on repeated measurements (< 10%CV); however, there is less consistency between assays [62, 63]. Using these methods, several studies report that total CSF aSyn levels are lower in PD patients than normal controls or patients with other non-degenerative neurological diseases [45, 64–66]. In two meta-analyses, the sensitivity and specificity for distinguishing PD samples from normal controls were 72% and 88%, and 65% and 40% with modest positive predictive values and area under the curve values [67, 68]. The majority of these studies have been cross-sectional studies performed in early- to mid-stage, clinically defined PD (Table 1), although lower levels of CSF total aSyn is noted in prodromal PD as well in some patients with RBD and hyposmia [69]. While statistically significant group differences are observed, there is substantial overlap in individual values of PD patients and healthy controls, which limits the use of CSF total aSyn currently as a diagnostic tool. Furthermore, lower average levels of CSF total aSyn compared to controls are also found in DLB, progressive supranuclear palsy, and multiple systems atrophy as well which would make current assays of limited value in the differential diagnosis of parkinsonism [68, 70–72]. As PD progresses, aSyn levels in some patients rise and there is some correlation between these higher CSF aSyn levels and cognitive and motor dysfunction [46, 73–77], but this finding is not universal [60]. However, higher levels of CSF aSyn are also noted in Alzheimer’s disease, other neurodegenerative disease, such as Creutzfeldt Jakob disease [78–82], suggesting levels of this analyte degenerating synapses/neurons. Therefore, it is difficult to disentangle which processes may be PD-specific, and which may be due to non-specific neurodegeneration. As far as the association of CSF total aSyn and specific disease features, some studies have documented lower amounts of CSF total aSyn in non-tremor-dominant phenotypes than tremor dominant PD cases [45, 46, 60]. PD patients with RBD have higher CSF total aSyn than PD patients without RBD [83]. There are conflicting reports about whether lower [45] or higher [73, 75] CSF total aSyn relates to worse cognitive outcomes in PD. In summation, on average, CSF total aSyn is lower in PD than healthy controls, especially early in the disease course but there is significant overlap of CSF total aSyn levels with healthy controls and other neurological diseases and therefore current assays cannot acceptably function as a single test to aid in the diagnosis of PD. Ratios of CSF total aSyn and other analytes discussed below may offer some improvement in diagnostic utility [64, 80], but more work, especially longitudinal measurements are needed to clarify the utility of this biomarker in PD in relations to other conditions..

**Table 1:** Selected studies of CSF alpha-synuclein in PD

Study	Biomarker	Subjects	PD duration	Assay specifics	Group comparisons	Clinical correlates
Parnetti et al. [64]	t-aSyn o-aSyn Abeta42 p-tau t-tau	PD: 44 OND: 25	Median 3 years (1–5.25 IQR)	aSyn ELISA: aSyn211/ aSyn FL-140 ELISA for Abeta42, t-tau, p-tau (Innotest AlzBio3 Fujirebio)	t-aSyn: ↓PD v OND Sns: 0.59, Spc: 0.80 o-asyn: ↑PD v OND Sns: 0.89, Spc:0.48 o-aSyn/t-Asyn: ↑PD v OND Sns: 0.82, Spc: 0.64 Abeta42/t-tau: PD↑ v OND Sns:0.82, Spc:0.56	Lower Abeta42 correlated with worse performance on MMSE and MoCA Abeta42/t-tau correlated with worse performance on MMSE
Kang et al. [45]	t-aSyn Abeta42 p-tau t-tau	PD: 412 HC: 189 SWEDD: 59	4.2y (range 0.03–35.8)	aSyn ELISA: aSyn 118–123/ aSyn 103–107 (Covance) ELISA for Abeta42, t-tau, p-tau (Innotest AlzBio3 Fujirebio)	t-asyn: ↓PDv HC t-tau: ↓PD v HC p-tau: ↓PD v HC Sensitivity, specificity, AUC not reported	T-aSyn↓ in non-TD than TD PD subjects Lower t-aSyn associated with worse cognition Lower Aβ1-42 associated with worse: olfaction, semantic fluency, DAT uptake
Hong et al. [65]	t-aSyn DJ-1	PD: 117 HC: 132 AD: 50	8.1y ± 6.5	Anti aSyn Luminex assay Capture antibodies: anti aSyn LB509, 211, anti DJ-1	t-aSyn ↓PD v HC and AD Sns: 0.94, Spc:0.50	Higher t-tau associated with worse cognitive testing scores
Tokuda et al. [66]	t-aSyn	PD: 33 OND: 29 HC: 9	63.4y ± 11SD	aSyn ELISA: aSyn 211/ aSyn FL-140	t-aSyn ↓PD v OND and HC. AUC 0.874 t-aSyn declines with age for all groups at similar rates	Lower CSF aSyn associated with worse HY score
Hall et al. [73]	t-aSyn Abeta42 t-tau p-tau CSF NfL	PD: 42 HC: 69	Median 7y (IQR 4–10.3)	Luminex xMap assay anti aSyn 9B6/ anti aSyn 4D8	t-aSyn, t-tau, p-tau ↓PD v HC	Lower CSF Abeta42 and higher CSF aSyn related to worse cognitive decline Higher CSF aSyn and p-tau related to worse motor decline
Stewart et al. [75]	t-aSyn	PD: 304	2.1y ± 1.4 and 3.8y ± 1.5 (phase 1 and 2)	Luminex assay Capture antibodies: anti aSyn LB509, 211	See clinical correlates	Higher t-aSyn associated with worse cognitive performance longitudinally t-aSyn decreased over time No association between t-aSyn and motor scores
Irwin et al. [46]	t-aSyn Abeta42 t-tau p-tau	PD: 416 HC: 192	6.7y ± 6.5	aSyn ELISA: aSyn 118–122/ aSyn 103–108 (Biolegend) ELISA for Abeta42, t-tau, p-tau (Innotest AlzBio3 Fujirebio)	t-aSyn Abeta42, t-tau and p-tau ↓PD v HC No Sns, Spc reported Abeta42 decreases over time P-tau and t-tau increase over time but with similar slopes in PD and HC. No longitudinal aSyn	Decreases in Abeta42 and increases in p-tau associated with worse cognitive outcome Lower Abeta42 and lower t-aSyn associated with increased UPDRSIII scores and PIGD subscores
Majbour et al. [76]	t-aSyn o-aSyn p-aSyn Abeta40 Abeta42 t-tau p-tau	PD: 121	Median 0.6y (IQR 0.4–0.9)	aSyn ELISAs: t-aSyn: aSyn-140/ 11D12 p-aSyn: aSyn-140/ pS129 o-aSyn: aSyn-O2/ FL-140 ELISA for Abeta42, t-tau, p-tau (Innotest AlzBio3 Fujirebio)	t-aSyn and o-aSyn increased and p-aSyn decreased over 2 years	Weak positive correlation of o-aSyn/total aSyn and UPDRS III scores and axial subscores
Goldman et al. [60]	t-aSyn Plasma aSyn Saliva aSyn Abeta42 t-tau p-tau	PD: 115 HC: 88	8.3y ± 3.1	aSyn ELISA: aSyn 118–122/ aSyn 103–108 (Biolegend) ELISA for Abeta42, t-tau, p-tau (Innotest AlzBio3 Fujirebio)	CSF t-aSyn ↓PD v HC Saliva and plasma aSyn PD = HC CSF Abeta42 ↓PD v HC	t-aSyn lower in PIGD CSF Abeta42 correlated with worse MoCA scores
Hall et al. [77]	t-aSyn Abeta42 p-tau t-tau YKL40 and NfL	PD: 63 HC: 21	5.5y ± 4.0	aSyn ELISA: aSyn 118–123/ aSyn 103–107 (Covance) ELISA for Abeta42, t-tau, p-tau (Innotest AlzBio3 Fujirebio) NfL (Uman Diagnostics) YKL-40 Quantikine ELISA Kit (R&D)	t-aSyn, t-tau, p-tau, NfL, and YKL-40 increases over 2 years in PD No major differences between PD and controls at baseline or 2 years	Increased p-tau and YKL-40 associated with worse cognitive decline, Increased p-tau also associated with worse motor decline No other correlations with Motor or cognition. NfL related to disease duration
Compta et al. [84]	t-aSyn o-aSyn	PDND: 21 PDD: 20 RBD: 23 HC: 13	Median 9.5y (IQR 64–76)	aSyn ELISA: t-aSyn: KHB0061 (Invitrogen) o-aSyn: anti aSyn 211/ 211	o-aSyn ↑ in PDND and PDD v RBD and HC t-aSyn no differences	o-aSyn with a positive correlation for UPDRIII and negative correlation with MMSE
Stewart et al. [86]	p-aSyn	PD: 304 LRRK2: 30	1.9y ± 1.4 in DATATOP subjects 7.9y ± 6.5 in UW collaborative study subjects	Luminex Assay ASY-1/pSer129	p-aSyn increased over 2 years, t-aSyn slightly decreased	Higher p-aSyn associated with lower UPDRSIII scores early but associated with higher UPDRSIII scores later in disease
Mollenhauer et al. [71]	t-aSyn Abeta42 t-tau	PD: 51 DLB: 55 MSA: 29 AD: 62 OND: 76	PD 12.2y ± 5.4	ELISA t-aSyn mSA1/Syn-1BB ELISA for Abeta42, t-tau, (Innotest AlzBio3 Fujirebio)	t-aSyn ↓PD v HC, AD PD = DLB and MSA	
Chahine et al. [116]	t-aSyn plasma t-aSyn	PD: 59 HC: 21	4.8y ± 4.6	ELISA t-aSyn and plasma aSyn aSyn 118–122/ aSyn 103–108 (Biolegend)	CSF t-aSyn ↓PD v HC Sns 0.87, Spc 0.63 AUC 0.69 Plasma t-aSyn PD = HC	
Schulz et al. [87]	t-aSyn p-aSyn t-tau NfL Serum aSyn others	PD: 151 DLB: 45 MSA: 17 PSP: 38 AD: 11 CBS: 16 FTD/ALS: 15 HC: 20	Not recorded	ELISA t-aSyn aSyn 118–122/ aSyn 103–108 (Biolegend) p-aSyn Erenna Immunoassay system SIMOA tau, NfL (Quanterix)	t-aSyn ↓PD DLB MSA PSP and CBS v HC No differences in p-aSyn or serum aSyn AUC PD v HC t-aSyn 0.746 p-aSyn 0.604 serum aSyn 0.564	No correlations of t-aSyn with UPDRS or HY ↑p-aSyn associated with worse MMSE

*t-aSyn* total alpha-synuclein, *o-aSyn* oligomeric alpha-synuclein, *p-aSyn* phosphorylated alpha-synuclein, *t-tau* total tau, *p-tau* phosphorylated tau, *HC* healthy controls, *OND* other neurological disease, *AUC* area under curve, *DLB* dementia with Lewy bodies, *MSA* multiple systems atrophy, *PSP* progressive supranuclear palsy, *CBS* corticobasal syndrome, *FTD/ALS* frontotemporal dementia/amyotrophic lateral sclerosis, *HY* Hoehn and Yahr scale, *UPDRS* Unified Parkinson’s disease rating scale, *MMSE* Mini Mental Status Exam, *ELISA* enzyme linked immunosorbent assay, *SIMOA* single molecule array

Other CSF aSyn species that have been studied in PD include phosphorylated and oligomeric forms of aSyn [75, 84]. Phosphorylated aSyn assays have focused on the pSer129 epitope which is a well described post-translational modification acidic tail near the C-terminal end of the protein in Lewy pathology [85]. In some studies, there is a U-shaped associated with disease severity with lower phosphorylated aSyn being associated with worse initial clinical presentations but later, higher levels being associated with worse motor and cognitive function [84, 86, 87].

The precise species of aSyn which contributes to neuronal dysfunction and neurodegeneration is not entirely clear, but the formation smaller oligomeric aggregations of aSyn may confer damage to synapses [88]. Higher levels of oligomeric aSyn from CSF samples compared to AD and healthy controls has been noted in PD and DLB [89]. Thus, there may be PD-specificity for oligomeric aSyn species detected in CSF as opposed to total aSyn measurements summarized above. Early studies suggest correlations of levels with CSF oligomeric aSyn with motor symptoms and may be useful as part of an oligomeric/total CSF aSyn ratio but replication in other laboratory settings is needed [90–93]. Early work in these studies use immunoassays which relay on epitope-specificity of the capture antibody used to detect oligomeric confirmations of aSyn. More recently, a study using a newer technique of single molecule counting technology was unable to detect pSer129 aSyn in CSF samples from PD patients, raising questions about epitopes specificity of immunoassays for different forms of aSyn in CSF [94]. Thus, these assays remain exploratory for PD research at the moment until further validation is performed.

## Plasma aSyn Measurements

Plasma aSyn measurements in PD have yielded differing results, with most studies reporting higher plasma total aSyn levels than healthy controls [13, 95–101], but other report no difference [102, 103] and still others reporting lower amounts in PD patients compared to controls [14, 60, 104]. There also similarly remains substantial overlap in the ranges observed between PD patients and healthy controls, which make would make plasma aSyn difficult to use as a single diagnostic test for PD. Similar to CSF assays, these studies use different capture and detection antibodies and this and other sources of variability may influence results (aSyn levels in red blood cells from hemolysis, age variation, etc.). Initial studies largely have used ELISA-based assays but more sensitive assays like single molecule arrays or immunomagnetic reduction assays may provide improved clarity on these relationships with both diagnosis and clinical features [13, 14, 95–97, 101] (Table 2). There is conflicting evidence about whether increasing levels correlate with worse motor dysfunction [60, 95, 100, 102, 105, 106], but higher levels have been reported to be associated with worse cognitive function [13, 95, 101, 102]. One longitudinal study had noted an increase in plasma total aSyn over time in PD patients [106]. Heterogeneity in cohorts, sample size and methodological issues of sample collection and analysis could contribute to conflicting results across studies, necessitating further studies in large multicentered cohorts using standardized operating procedures. Future longitudinal studies in deeply phenotyped cohorts will provide further clarity on the use and evolution of plasma aSyn biomarkers although some likely changes over time can be surmised from the prior studies (Table 3).

**Table 2:** Selected studies of plasma alpha-synuclein in PD

Study	Biomarker	Subjects	PD duration	Assay specifics	Group comparisons	Clinical correlates
Chen et al. [95]	t-aSyn, Abeta42, Abeta40, t-tau	60 PD 28 HC	median 1y (IQR 0–3)	Immunomagnetic reduction-based immunoassay (MF-ASC-0060)	t-aSyn ↑PD v HC	↑plasma aSyn correlated with: ↑UPDRS, ↑HY, ↓MMSE ↑plasma t-tau correlated with: ↑UPDRS, ↑HY, ↓MMSE ↑plasma Abeta40 correlated with: ↓UPDRS, ↓HY, ↑MMSE
Ng et al. [13]	t-aSyn	170 PD 51 HC	5.0y ± 5.0	SIMOA (Quanterix) Antibodies not disclosed	t-aSyn ↑PD v HC	No change in HY. PD with UPDRS III > 24 or with MMSE ≤ 25 had higher t-aSyn than controls
Chang et al. [96]	t-aSyn serum aSyn	88 PD 40 HC	9.1y ± 6.5	Immunomagnetic reduction assay aSyn 121–125	Plasma and serum t-aSyn ↑PD v HC AUC 0.99, .92	Serum aSyn correlated with HY 1–3 but not plasma aSyn
Youssef et al. [62]	t-aSyn	49 PD 47 HC	4y ± 0.3	ELISA aSyn 118–122/ aSyn 103–108 (Biolegend) ELISA aSyn 110–125/ unmapped detection aSyn Ab (15–125) (Mesoscale Discovery) SIMOA ADx301/ ADx302 (Quanterix)	t-aSyn ↑PD v HC for all assays albeit at different degrees SIMOA assay detecting 8–10 × more plasma aSyn than other assays in both HC and PD cases R = 0.62–0.67 p < 0.0001 for correlations between assays	No correlations with age at diagnosis, disease duration, HY score
Foulds et al. [106]	t-aSyn and p-aSyn	189 PD 91 HC	5.1y ± 4.1	ELISA t-aSyn aSyn 211/ aSyn FL-40 p-aSyn aSyn N19/ aSyn pSer129	t-aSyn: trend towards ↑PD v HC (p = 0.058) at baseline t-aSyn increases over time p-aSyn ↑PD v HC at baseline no change over time	
Duran et al. [98]	t-aSyn	53 PD untreated 42 PD treated 60 HC	PD untreated: 0y PD treated: 9.2 ± 1.1	ELISA Invitrogen KHB0061	t-aSyn ↑ PD v HC t-aSyn = PD untreated v PD treated	
Lee et al. [99]	t-aSyn	105 PD 38 MSA 51 HC	PD 3.7y ± 3 MSA 3.9y ± 2.3	ELISA aSyn 117–131/ ‘rabbit aSyn antibody’	t-aSyn ↑ PD v HC t-aSyn ↑ MSA v HC Plasma aSyn ↑ PD v MSA	No correlations with age or HY
Gorostidi et al. [104]	t-aSyn, o-aSyn	134 PD 32 PD LRRK2 109 HC	PD 6.2 ± 5.3 PD LRRK2 7.5 ± 6.3	ELISA t-aSyn aSyn 211/ aSyn FL-140 ELISA o-aSyn aSyn 211/ aSyn 211	t-aSyn ↓ in PD v HC t-aSyn = in PD LRRK2 v HC	No correlations with age, disease duration, HY
Li et al. [14]	t- aSyn	13 EO PD 14 LO PD 11 HC	> 5y	Quantitative WB aSyn 97/8	t-aSyn ↓ EO and LO PD v HC	
Caranci et al. [102]	t-aSyn	69 PD 110 HC	10.8y ± 7.3	ELISA Invitrogen KHB0061	No difference in t-aSyn between PD and HC or PD men v women	↓ t-aSyn associated with worse HY, UPDRS scores, and cognitive status in men only
Fan et al. [100]	t-aSyn NLRP3	43 PD 24 HC	2.3y ± 0.3	UPlex Assay (Mesoscale Discovery) No information on antibodies	t-aSyn ↑PD v HC t-aSyn correlates with IL-1β	↑ t-aSyn associated with worse UPDRSIII scores
Lin et al. [101]	t-aSyn	80 PD 34 HC	7.5y ± 5.2	Immunomagnetic reduction aSyn211/ aSyn1-140	t-aSyn ↑PD v HC	↑ t-aSyn PDD > PD with normal cognition and with higher HY stage
Shim et al. [103]	t-aSyn	20 PD 20 HC	Not reported	ELISA 4B12/ 4D6	t-aSyn PD = HC t-aSyn correlates with hemolysis	No correlation of aSyn with HY or age

*t-aSyn* total alpha-synuclein, *o-aSyn* oligomeric alpha-synuclein, *p-aSyn* phosphorylated alpha-synuclein, *t-tau* total tau, *p-tau* phosphorylated tau, *HC* healthy controls, *OND* other neurological disease, *AUC* area under curve, *HY* Hoehn and Yahr scale, *UPDRS* Unified Parkinson’s disease rating scale, *MMSE* minimental status exam, *ELISA* enzyme linked immunosorbent assay, *SIMOA* single molecule array, *EO PD* early onset PD, *LO PD* late onset PD, *LRRK2* leucine rich repeat kinase 2, *PDD* parkinson’s disease dementia

**Table 3:** Changes in biomarkers compared to healthy control populations. As PD progresses, there is heterogeneity in the change in biomarker profiles such that some patients may increase or decrease in certain biomarkers, but others may remain stable (↑ = or ↓ =). There are competing articles about the changes seen in oligomeric aSyn over time (↓↑)

Biomarker	Prodromal PD	Early PD	Mid/late PD
CSF			
t-aSyn	↓ =	↓	↑ =
o-aSyn		↑	↑↓
p-aSyn		↑	↑
Aβ42		↓	↓ =
t-tau		↓	↑ =
p-tau		↓	↑ =
Plasma			
t-aSyn		↑	↑
Skin IF	+	+	+
aSyn-SAA	+	+	+

*t-aSyn* total alpha-synuclein, *o-aSyn* oligomeric alpha-synuclein, *p-aSyn* phosphorylated alpha-synuclein, *IF* immunofluorescence, *aSyn-SAA* alpha-synuclein seeding amplification assay

## ASyn Immunohistochemistry and Immunofluorescence from Tissue Samples

aSyn deposits are found in autonomic nerves that innervate a variety of peripheral tissues including, skin, olfactory mucosa, submandibular glands, and the colon in PD [16, 107–110]. Thus, the presence of these phosphorylated deposits in peripheral tissues could potentially aid the tissue diagnosis of living PD and other synucleinopathies.

Given that aSyn deposits likely occur early in the dorsal motor nucleus of the vagus nerve and the olfactory bulb, Heiko Braak and others posited that environmental factors could cause aSyn changes that could propagate to the central nervous system either from the gut via the vagus nerve or from the olfactory epithelium [111, 112]. Moreover, model systems find evidence to suggest pathological aSyn can propagate from the gut to the brain, which is abolished by vagotomy [113]. However, human autopsy studies do not find clear evidence of “incidental” peripheral aSyn in tissues (i.e., isolated aSyn in peripheral tissues without involvement of the brain), which argue against a peripheral origin of aSyn in PD and related synucleinopathies and instead peripheral aSyn may spread from early brainstem pathology [114]. It is very difficult to definitively define the epicenters or origins of neurodegenerative pathologies using cross-sectional autopsy tissue alone, but the findings of peripheral aSyn in PD and DLB offer an important minimally invasive method to obtain tissue diagnosis in living patients.

Given the regularity of colonoscopies as screening tests for colon cancer, investigations at assessing for aSyn pathology in colonic biopsies were performed but initial results were highly discordant with varying sensitivities in detecting pathological aSyn deposits in PD patients [115–117]. Multi-site studies were performed that showed good inter-rater reliability and helped to optimize methods, but results indicate that biopsies must include submucosal layers and be assessed by trained neuropathologists to determine if adequate neuronal elements and aSyn deposits are present [118, 119]. The submandibular glands of PD patients also demonstrate aSyn inclusions but there is a higher morbidity associated with needle biopsies compared to other peripheral tissue sampling and, because of inadequate sampling and immunohistochemical methods, sensitivity remains suboptimal in many studies [110, 116, 120–122]. aSyn deposits in skin biopsies are currently the most promising and least invasive tissue-based biomarker with optimization of methods that has occurred over the last several years. Initial studies discovered that different biopsy sites could yield different sensitivities in detecting phosphorylated aSyn deposits, with the abdomen and scalp showing lower rates of positivity but higher rates being shown in paracervical and lower leg sites in PD and DLB patients [16, 107–109, 123–125]. Different fixative methods influence results, with formalin fixed paraffin embedded tissue not performing as well as Zamboni fixation methods [15, 116, 124, 126–132]. The main reason for this may be that formalin may cause more extensive protein cross linking, making it more difficult for antibodies to attach to aSyn epitopes and the heat or chemically based retrieval methods may decrease aSyn signal if used too aggressively [116, 133, 134]. Depth of biopsy and section thickness affects yield as well [133]. Immunofluorescence using double labelling with antibodies against phosphorylated aSyn and neuron specific protein gene product (PGP) appears to perform better than bright field immunohistochemistry using diaminobenzamide chromagen (DAB) [15, 123, 127, 129–131, 135, 136]. The DAB chromagen is a staple of immunohistochemistry and creates a dark brown signal when detecting epitopes. However, in skin samples, it can be difficult to discern DAB signal from artifact and diffuse non-specific staining in small peripheral nerves and immunofluorescence facilitates double labelling to identify the overlap of small neurons innervating the skin and the presence of small phosphorylated synuclein inclusions simultaneously in the same tissue section [134, 137]. The most current methodologies using Zamboni fixative, cryosectioning, and immunofluorescence show 90% sensitivity and > 90% specificity for PD and DLB subjects in some studies [15, 125, 132, 138, 139]. Thus, standardization of pre-analytical factors, including sample handling are critical for the development of these tests for clinical use [140].

Aside from clinically manifest PD, skin aSyn deposits can be demonstrated in patients with REM sleep behavior disorder (RBD) and patients with pure-autonomic failure (pAF), both thought to be prodromal states that are highly likely to phenoconvert into PD or other synucleinopathy where presumably central nervous system pathology is more restricted [123, 126, 131, 137, 141–144]. It is not clear yet whether a positive skin biopsy predicts phenoconversion in RBD subjects but studies with longitudinal follow up are underway [137, 145]. Interestingly, there also may be differences in the characteristics of aSyn deposits between MSA and PD patients where MSA patients had phosphorylated aSyn deposits in somatic nerves whereas PD patients with orthostatic hypotension had deposits in autonomic nerve fibers in one study [146]. Furthermore, in PD patients, aSyn positive skin biopsies appear to have a rostro-caudal gradient, with more positive samples being noted from paracervical biopsy sites than limb sites; in MSA, however, there is a more uniform distribution of aSyn positivity in the different biopsy sites and higher density of phosphorylated aSyn in those biopsies [147]. Orthostatic hypotension is a common but not universal symptom of PD and its presence signifies autonomic involvement which may have relevance for skin biopsies in PD [148, 149]. One study found that PD patients with orthostatic hypotension had a more widespread and homogenous distribution of aSyn deposits whereas PD patients without orthostatic hypotension has aSyn pathology restricted to paracervical biopsy sites [150]. While these biopsies are likely useful in a categorical fashion, there are no features that correlate well with disease severity; however, one study of an MSA patient who underwent serial skin biopsies did note sequentially more skin structures affected, implying an evolution of skin aSyn deposits over time [146, 151]. aSyn skin deposits from PD patients with *LRRK2*, *GBA*, and *SNCA* mutations have also been demonstrated [152–155]. Given the pathological heterogeneity associated with *LRRK2* mutations and the limited degree of central nervous system aSyn deposits in patient with *PRKN* mutations, further studies in autopsy validated subjects will be of interest [156, 157]. See Table 4 for selected studies of skin aSyn immunofluorescence in PD.

**Table 4:** Selected studies in aSyn-SAA and skin immunofluorescence/immunohistochemistry

Study	Assay	Sample	Subjects	PD duration	Results
aSyn-SAA					
Fairfoul et al. [17]	0.1 mg/ml rec aSyn WT (Stratech)	BH and CSF	Discovery: DLB:29 PD:2 AD:30 PSP:2 CBS:3 ILBD:13 HC: 20 Validation: PD:20 HC:15 RBD: 3	Not provided	DLB v HC: Sns 0.92, Spc: 1.00 PD v HC: Sns 0.95, Spc: 1.00 3/3 RBD patients + aSyn-SAA
Shahnawaz et al. [19]	1 mg/ml rec aSyn WT + 6hist (local)	BH and CSF	PD: 76 OND: 65 NDG: 18 AD: 14	Not provided	PD v disease controls: Sns: 0.89 Spc: 0.94 Time to reach 50% maximum aggregation inversely correlated with HY stage
Groveman et al. [20]	0.1 mg/ml rec aSyn K23Q + 6hist (local)	BH and CSF	PD: 12 DLB: 17 Non-aSyn: 31	2.9y	PD and DLB v non-aSyn: Sns: 0.93, Spc: 1.00
Bargar et al. [174]	1 mg/ml rec aSyn WT (rPeptide)	BH, CSF, Saliva, Skin, colon	PD: 88 DLB: 58 Controls: 68	Not Provided	CSF: PD and DLB v controls: Sns 0.98, Spc 1.00 Sns and Spc not analyzed for other tissues
Iranzo et al. [169]	0.1 mg/ml rec aSyn WT (Sigma)	CSF	RBD: 52 HC: 40	NA	RBD v HC: Sns 0.90 Spc 0.90 During 7y follow up 32 photoconverted to PD or DLB (31/32 + aSyn SAA)
Siderowf et al. [197]	0.3 mg/ml rec aSyn WT + 6hist	CSF	PD: 545 HC: 163 SWEDD: 54	Sporadic PD 0.6y ± 0.5 LRRK2 PD 3.0y ± 2.1 GBA PD 3.5y ± 2.4	All PD cases v HC: Sns 0.88 Spc: 0.96 Sporadic PD v HC: Sns: 0.93, Spc: 0.96 LRRK2 PD v HC: Sns: 0.68, Spc: 0.96 GBA PD v HC: Sns: 0.96, Spc 0.96
Rossi et al. [168]	0.1 mg/ml rec aSyn WT	CSF	Clinical RBD: 18 PAF: 28 PD: 71 DLB: 34 OND: 135 Path-validated LB + : 21 LB-: 101	Clinical PD: 56.8 m ± 45.8	Neuropathologically validated cases with aSyn Sns: 0.95 Spc: 0.98 Clinical diagnoses aSyn v OND: Sns: 0.95, Spc 0.98 18/18 RBD + aSyn SAA, 26/28 PAF + aSyn-SAA
Russo et al. [167]	AbbVie RT-QuIC: 0.1 mg/ml rec aSyn WT (local), Caughey RT-QuIC: 0.1 mg/ml rec aSyn K23Q PMCA: 0.3 mg/ml rec aSyn WT + 6hist	CSF	PD: 30 HC: 30 SWEDD: 20	PPMI: 6.7 m ± 6.5 SAA: 9.0 m ± 8.4	PD v HC at baseline: AbbVie: Sns:0.89, Spc: 1.00 Caughey: Sns: 0.86, Spc: 0.97 Amprion: Sns: 0.96, Spc: 0.97 PD v HC Year 3 AbbVie: Sns: 0.93, Spc: 0.93 Caughey: 0.89, Spc: 0.97 Amprion: 0.96, Spc: 0.93
Poggiolini et al. [170]	1 mg/ml rec aSyn WT	CSF	PD: 74 MSA: 24 RBD: 45 HC: 55	PD 2.1y ± 1.4y	PD v HC: Sns: 0.89 Spc 0.96 No major correlations of kinetic parameters and clinical features in PD MSA v HC: Sns: 0.75 Spc 0.96 (longer T50, lower Fmax) Some correlations of kinetic parametiers and clinical features in MSA RBD v HC: Sns: 0.64 Spc 0.96 14/45 phenoconverted in the 0.2–7.9 y of followup. 9/14 + aSyn SAA at baseline
Kang et al. [166]	RT-QuIC: 0.1 mg/ml rec aSyn WT (Sigma) PMCA: 0.3 mg/ml rec aSyn WT + 6hist	CSF	PD: 105 HC: 79	8 (4–17)	RT-QuIC: PD v HC Sns: 0.95 Spc: 0.96 PMCA: PD v HC Sns 0.90 Spc: 0.82
Kuzkina et al. [172]	RT-QuIC Cleveland aSyn rec WT (rPeptide) RT-QuIC Wurzburg 5 mg/ml aSyn rec WT (in house)	Skin: 5 mm. C7, T12, thigh, lower leg	PD: 34 HC: 30	11.7y ± 6.9	Sns: 0.91, Spc 0.87 Κ = 0.86 for patient results between labs
Manne et al. [21]	0.1 mg/ml aSyn rec WT	Skin	Frozen PD: 25 HC: 25 Formalin fixed PD: 12 HC: 12	Not described	Frozen Sns 0.96 Spc 0.96 Fixed Sns: 0.75 Spc: 0.83
De Luca et al. [177]	5 mg/ml aSyn rec WT	Olfactory Mucosa	PD: 18 MSA: 11 OND: 18	10.1y ± 5.1	PD Sns: 0.56 MSA Sns: 0.82 Spc: 0.83
Skin IF/IHC					
Donadio et al. [138]	IF Zamboni pSer129 aSyn/PGP Cryosectoining CSF and skin RT-QuIC: 0.1 mg/ml rec aSyn WT (rPeptide)	3 mm C7, thigh, leg CSF	IF reproducibility PD: 4 MSA: 4 DLB: 1 OND: 12 IF v RT-QuIC PD: 17 DLB: 5 MSA: 8 PAF: 3 OND: 38 HC: 24	Not available	aSyn v non aSyn Skin IF: Sns: 0.90 Spc: 1.00 CSF RT-QuIC: Sns 0.78 Spc 1.00 Skin RT-QuIC: Sns 0.86 Spc 0.80
Gibbons et al. [15]	IF: Zamboni, pSer129 aSyn/PGP Cryosectioning	3 mm distal leg, proximal/distal thigh, forearm	PD: 28 HC: 23	PD nAF 4.3y ± 5.1 PD AF 8.6y ± 7.3	Sns 0.95 Spc 0.91
Wang et al. [133]	IF: Zamboni, pSer129 aSyn/PGP Cryosectioning	3 mm distal leg or distal/proximal thigh	PD: 29 HC: 21	5.5y ± 5.1	50 µm sections: Sns 1.00, 20 µm sections: Sns: .90, 10um sections: Sns:73 Spc: 1.00
Donadio et al. [124]	IF: Zamboni pSer129 aSyn/PGP Cryosectioning	3 mm C7 2 × or C7 and T12	PD: 28	15 patients unilateral symptoms 3y ± 2 13 patients bilateral symptoms 10y ± 6	Sns 1.00 from C7 Sns 0.62 from T12 site No differences in laterality in spite of lateralized motor symptoms
Donadio et al. [132]	IF: Zamboni pSer129 aSyn, PGP Cryosectioning	3 mm C8, thigh, distal leg	PD: 21 Other Parkinsonism: 20 HC: 30	PD: 13y ± 6	Sns 1.00 Spc 1.00
Doppler et al. [126]	IF: PFA 4% pSer129 aSyn PGP Cryosectioning	5 mm Proximal and distal leg, T12, C7	PD: 25 RBD: 18 HC: 20		PD Sns: 0.80, RBD Sns: 0.56 Spc:1.00
Doppler et al. [136]	IF: PFA 4% pSer129 aSyn, PGP. Cryosectioning	5 mm, proximal and distal leg, T12, finger	PD: 31 HC 35	9.0y (range 0.3–27)	PD v HC Sns 0.52 Spc: 1.00
Al-Qassabi et al. [137]	IF: FFPE pSer129 aSyn/PGP	3-5 mm Leg or C8	PD: 20 RBD: 28 Other parkinsonism: 10 HC: 21	PD 8.4y ± 4.4	PD Sns 0.70, RBD Sns: 0.82, Other parkinsonism 0.20. Spc 1.00
Chahine et al. [116]	IHC: FFPE aSyn. Proteinase K	3 mm C7-8, mid thigh	PD: 58 HC: 21	4.8y ± 4.6	Sns 0.24 Spc 1.00

For aSyn-SAA studies, the type of aSyn used for reactions is detailed and for skin immunofluoresence/Immunohistochemistry, basic aspects of these assays are reported along with biopsy sites and type

*RT-QuIC* real-time quaking induced conversion, *PMCA* protein misfolding cyclic amplification, *rec aSyn* recombinant alpha-synuclein, *WT* wild type, *6hist* histidine tag, *BH* brain homogenate, *CSF* cerebrospinal fluid, *PD* Parkinson’s disease, *DLB* dementia with Lewy bodies, *AD* Alzheimer’s disease, *PSP* progressive supranuclear palsy, *CBS* cortico basal syndrome, *HC* healthy controls, *OND* other neurological disorders, *NDG* other neurodegenerative diseases, *MSA* multiple systems atrophy, *Sns* sensitivity, *Spc* specificity, *HY* Hoehn and Yahr stage, *RBD* REM sleep behavior disorder, *PAF* pure autonomic failure, *IF* immunofluorences, *IHC* immunohistochemistry, *PFA* paraformaldehyde, *FFPE* formalin fixed paraffin embedded, *PGP* neuron specific protein gene product

Peripheral biopsy testing for PD is nearing clinical use as there is a commercially available aSyn skin biopsy assay, the Syn-One test (CND Life Sciences). The SynOne test suggests obtaining samples using 3 mm punch biopsy tools, Zamboni fixative and requires double-immunostaining thick cryosection for neuronal elements (PGP 9.5) and phosphorylated aSyn (pSer129) using immunofluorescence [158]. Unpublished data using the Syn-One test has been presented at the American Academy of Neurology meeting in 2020 and the Lewy Body Disease association Biofluid/Tissue Biomarker symposium in 2021 reporting high sensitivity (74% from one biopsy site and 96% from three biopsy sites) and 99% accuracy of distinguishing synucleinopathies from controls [159]. This test is not FDA approved but is being further validated in a large multicentered clinical trial (NCT04700722) with a plan to enroll over 300 patients with synucleinopathies (PD: 105, MSA: 40, DLB: 90, pure autonomic failure: 65) and 200 healthy controls who will undergo three skin biopsies at the paracervical, distal thigh, and lower leg sites to determine sensitivity, specificity, accuracy, and precision of the current test [158].

## Alpha Synuclein Seeding Amplification Assays

**Fig. 1:**
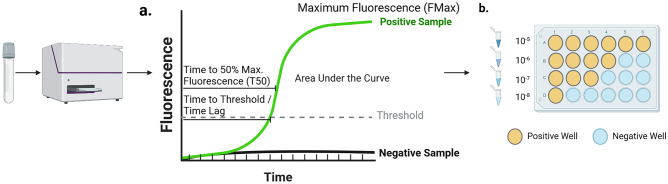
aSyn-SAA metrics. Tissue or fluid samples are analyzed with fluorescence measurements read at given intervals which can be used to establish curves shown in (**a**). From these curves, a variety of metrics can be derived including maximum fluorescence, time to threshold (or time lag), time to 50% of maximum fluorescence (T50) or area under the curve calculations; however, these metrics have not consistently been shown to relate to clinical characteristics or pathological burden within PD patients. If a sample undergoes serial dilution and is analyzed at these different dilutions as shown in (**b**), the dilution at which 50% of well remain positive can be used to estimate the SD50 which may have more relevance to disease activity in some studies. In this example the estimated -log(SD50) = 7. Created with Biorender.com

aSyn seeding amplification assays (aSyn-SAA) began as adaptations of prion disease assays and make use of the ability of aSyn seeds to template normal monomeric aSyn species to oligomeric and fibrillar forms in a prion-like fashion [160–162]. In these assays, a biological sample is added to a well containing monomeric aSyn with a fluorescent tag thioflavin-T. If a pathological aSyn seed is present, it will induce templating of the monomers and after a certain amount of time, the newly created fibrils will be broken down by shaking the plate allowing for more monomers to be recruited. After several hours, this creates an exponential rise in the fluorescence which can be detected. The standard diagnostic metrics collected is a binary positive or negative readout above a certain fluorescence threshold defined by the laboratory, but additional metrics including the time to positive signal (or lag time), maximum fluorescence, and the time to reach 50% of maximum fluorescence can also be reported if fluorescence measurements are captured at regular intervals (Fig. 1). In initial studies, remarkably high sensitivity and specificity (> 90%) was demonstrated in detecting aSyn seeding from CSF samples of patients with manifest PD and DLB [17, 163]. In the years since, multiple studies in independent laboratories have confirmed these findings [18, 20, 160, 163–166]. aSyn seeds are readily apparent in early PD when subjects within 2 years of diagnosis who had not started medications from the Parkinson’s Progression Marker Initiative were studied [166, 167], and high rates of positivity are also observed in prodromal patients with REM sleep behavior disorder and pure autonomic failure, conditions which have a high likelihood of underlying alpha-synuclein and phenoconverting into PD or DLB [165, 168–170]. In the case of REM sleep behavior disorder, it is not entirely clear if a positive aSyn-SAA results predicts phenoconversion to one of these syndromes. Some of the uncertainty is due to lack of longitudinal studies with serial sampling and differences in baseline rates of aSyn-SAA positivity in RBD cohorts studied [169, 170].

This body of work suggests that these assays are extremely sensitive and specific for detecting the categorical presence/absence of aSyn seeds in CSF of patients with manifest disease and prodromal states where presumably aSyn pathology is more restricted. It is less clear whether the quantitative metrics collected by these assays have quantitative value in relation to clinical variables in PD. Aside from MSA, there are no major differences in maximum fluorescence, time to positivity, or area under the curve between PD, DLB, pure autonomic failure or REM sleep behavior disorder patients [165]. In the majority of studies conducted, there have been no strong correlations with time to positivity, maximum fluorescence or time to 50% fluorescence with clinical aspects of Parkinson’s disease such as motor burden [166, 167]. There was one study where mild to moderate correlations of time to threshold and maximum fluorescence were observed with disease duration and motor burden on the unified Parkinson’s disease rating scale but this has not been replicated [167]. While not routinely performed in clinical assays, serial dilution of biological samples can be used to calculate an SD50 value, the seeding dose at which 50% of wells will turn positive, using a Spearman-Karber method [171] (Fig. 1). In two studies, aSyn-SAA SD50 values correlated with disease duration and higher SD50 values were noted with higher degrees of pathological aSyn deposits in postmortem autopsy analyses [160, 167, 172]; however, it has not been consistently related to other disease features and such methods are time consuming and unlikely to be scaled for routine use. Regarding pure autonomic failure, subjects with this condition may phenoconvert to PD, DLB, or multiple systems atrophy (MSA) and there is some evidence that the kinetics of the aSyn-SAA curve (i.e., maximum fluorescence and time to positivity) and additional information from neurofilament light chain testing in CSF may offer prognostic information about which synucleinopathy a subject is likely to phenoconvert to [163, 165]. Samples from patients with MSA in some studies have a faster time to positive but lower maximum fluorescence and this may reflect properties of different aSyn strains in these associated diseases as numerous biochemical and structural differences between the aSyn species in MSA and Lewy body disorders have been described [163, 165, 168].

**Fig. 2:**
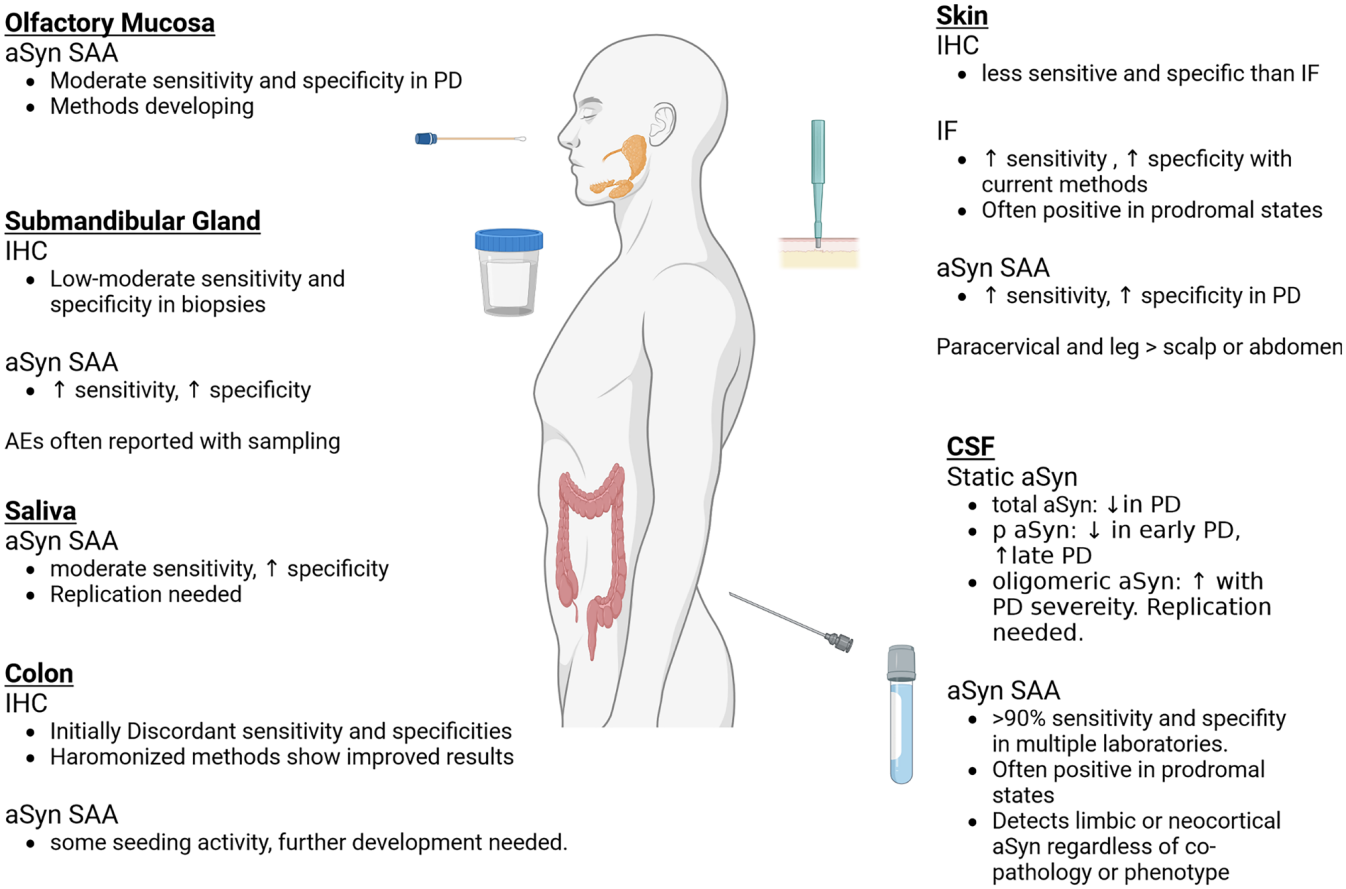
aSyn assays from biofluids and tissue. Summary of static aSyn assessments, peripheral tissue immunohistochemistry, immunofluorescence and aSyn-SAA assays in different tissues and fluids studied currently with spinal fluid aSyn-SAA and skin aSyn-SAA and immunofluorescence assays showing the greatest accuracy to date but with many other assays still in development. Created with Biorender.com

In the last few years, several attempts have been made to adapt aSyn-SAA assays from CSF above to peripheral tissue and fluid samples, which could potentially offer a less invasive manner of diagnosing the presence of aSyn seeds (Fig. 2). Much of this initial work was pursued because of the well documented observations of abnormally phosphorylated aSyn deposits in skin, colon, submandibular gland, and other tissues in PD patients both at autopsy and in vivo from biopsy studies discussed above [116, 134]. aSyn-SAA from skin biopsies appear to offer similarly high sensitivity and specificity comparable to CSF in several studies in PD and RBD patients [21, 172–176]. Olfactory mucosa samples may be useful as well, but sampling requires accessing very deep structures, often using a rigid scope and with an otolaryngologist operator, which may limit feasibility [177]. Seeding from olfactory mucosa samples in PD, MSA, DLB, and REM sleep disorder patients has been demonstrated though [174, 177–179]. aSyn seeding activity has also been demonstrated from not only submandibular gland biopsies but also from saliva itself [180–182]. Lastly, seeding activity can be demonstrated from colonic biopsies, where phosphorylated aSyn has been known to deposit [116, 134, 174, 183]. See Table 4 for selected aSyn-SAA studies.

The majority of the above studies have been performed in clinically defined cohorts, and in those studies where neuropathological confirmation has been performed, co-pathologies are not typically assessed in a standardized fashion [17, 160]. While aSyn aggregates in Lewy bodies and Lewy neurites are noted in brainstem, limbic and neocortical areas in PD and DLB, Lewy bodies, and Lewy neurites are present in the amygdala and nearby limbic structures in about 50% of sporadic Alzheimer’s disease patients and around 90% of familial Alzheimer’s disease cases with presenilin mutations [184–187]. Such cases are unlikely to exhibit PD or DLB like clinical phenotypes [24]. Two studies recently have addressed whether current CSF assays can detect aSyn seeds in these amygdala-predominant cases and both found that CSF assays detected aSyn seeds in these cases at much lower rates than in cases with limbic or neocortical stage Lewy pathology [188, 189]. Both studies also show that positivity of these assays is dependent on aSyn stage and not masked or significantly influenced by the co-occurrence of Alzheimer’s pathology [188, 189]. Direct seeding assays from frozen amygdala samples from amygdala-predominant cases also showed a mix of positive and negative reactions in one of these studies [189]. Further studies are needed to understand whether this variability in seeding activity is due to a lower overall dose of aSyn seeds or if there are differences in the aSyn species in amygdala-predominant cases that result in lower seeding activity. Such studies are important to understand the interpretation of aSyn-SAA results when applied to a larger population where subjects may harbor incidental Lewy bodies or amygdala predominant Lewy bodies. Furthermore, several population-based cohorts would suggest that the baseline prevalence of aSyn pathology is around 20–30%, and in some cases, this pathology can be widespread without causing clinical symptoms [23, 35, 190–193]. While it appears that these assays may be somewhat less sensitive to detect these cases of incidental Lewy body disease and amygdala-predominant disease, further studies will be needed [168, 188]. Additionally, some patients with (*LRRK2* mutations and most, if not all, patients parkin PBR E3 ubiquitin protein ligase (*PRKN*) will not have pathological aSyn accumulations at autopsy [156, 194] and therefore will be less likely to exhibit seeding activity or aSyn deposits [153, 195–197]. Therefore, there is likely a role of integrating genetic testing information into the application of these assays in PD.

At this time, there is also a commercially available CSF aSyn-SAA assay SynTAP (Amprion Laboratories) that is not FDA approved but did receive FDA breakthrough designation in 2019. In the SynTAP assay, which is a slightly modified version of Amprion’s research assay (formerly referred to as PMCA), samples are run in triplicate using glass beads with fluorescence measured less frequently than the research assay to allow for higher throughput [189, 198]. The SynTap assay has shown similarly high accuracy to Amprion’s research assay [189].

## AD Fluid Biomarkers in PD and DLB

As noted previously, autopsy studies of PD patients typically reveal 35–50% of PD patients with dementia and more than 70% of DLB patients have moderate to high levels of AD neuropathologic change [29–35]. AD co-pathology in PD has been associated with older age of onset, shorter disease duration, faster time to dementia, greater likelihood of amnestic memory deficits and greater likelihood of an akinetic rigid motor phenotype in several studies [31, 32, 36–42]. These findings are not universal however, and in cluster analyses of PD, no major changes in rates of AD co-pathology of CSF AD biomarkers in studies comparing so called diffuse-malignant subtypes of PD with mild motor-predominant forms [199, 200]. Still, understanding the interplay of aSyn, Aβ, and tau pathology in PD and DLB is of interest as it will inform the interpretation of AD biomarkers in these populations as these assays become more widely available and stratifying clinical trials by the presence or absence of AD co-pathology may be of interest [201].

PD and DLB patients tend to have lower levels of CSF Aβ42 and tau species than normal controls in groupwise comparisons early in the disease [45, 46, 49, 73, 202–204]. In PD, lower levels of CSF Aβ42 is related to worse cognition cross-sectionally, longitudinally, and is related to higher likelihood of AD co-pathology at death [44, 46, 47, 64, 73, 202, 204, 205]. Interestingly, one study showed an increase in CSF Aβ42 in PD patients with freezing of gait compared to PD patients who did not [206]; thus, clinical heterogeneity of PD may influence biomarker interpretation as well. While total and p-tau 181 is on average lower than controls in early PD, levels may increase later in the disease in some patients which is also associated with a greater likelihood of dementia [207–210]. While optimal cut-offs for these Aβ42, t-tau, and p-tau 181 and their ratios have been well established in Alzheimer’s disease, it is not clear if the same cutoffs apply in PD and other Lewy body disorders [211, 212]. Indeed, in rare autopsy-confirmed work, there is data to suggest CSF Aβ42 may be associated with increasing aSyn pathology independent of plaque burden in LBD [47].

More recently, plasma assays (Aβ1-42, t-tau, p-tau 181, p-tau 217, and p-tau 231) are being developed for use in AD but are already being studied in PD as well [213–216]. Plasma Aβ42 may be related to more severe gait impairment and severity of akinetic rigid symptoms [217, 218]. Plasma p-tau 181 and p-tau 217 levels correlate with degree of tau PET and Aβ PET status [219]. In studies of DLB, where tau co-pathology is more likely, plasma p-tau 181 and 231 have been associated with faster cognitive declines [219, 220]. Higher levels of plasma p-tau 181 are reported in PD patients when compared to healthy controls and these levels correlate with plasma aSyn markers [221]. However, in some studies plasma p-tau 181 has not clearly been linked to cognitive decline in PD and plasma t-tau and neurofilament light chain measurements have had stronger correlations with cognitive dysfunction [95, 105, 222]. In DLB, in particular, where rates of AD co-pathology are often quite high, stratification by the presence of these AD biomarkers may prove especially important for clinical trial enrollment of more biologically homogenous patients or those who may benefit from combination therapies [201].

## Conclusion

aSyn-specific biomarkers have long been an unmet need in the field of neurodegenerative medicine. While the search for biomarkers with strong associations with disease pathology continues, several new fluid and tissue based biomarkers are being developed which offer the ability to detect aSyn species in patients with PD, DLB, and also in prodromal states, which is critical for therapeutic trials targeting aSyn mechanisms. CSF aSyn and plasma aSyn species detected by current assays may be limited but further development with newer second-generation immunoassays or other methods of detection may provide additional opportunities for biomarker development. Please see Table 3 for a summary of CSF (Table 1), plasma (Table 2), and aSyn-SAA and immunofluorescence (Table 4) biomarker data findings in PD. aSyn immunofluorescence from skin samples and aSyn-SAA assays both from CSF and peripheral tissues appear promising and will likely be of imminent use in clinic and research settings which will likely provide accurate methods of categorically assessing for the presence of aSyn deposits and aSyn seeds [138]. More work will be needed to determine of more labor-intensive methods like calculating SD50 will provide quantitative readouts of aSyn seeding that have relevance for disease activity, but initial studies suggest some significant correlations with disease duration and pathological burden. Most studies of aSyn-SAA to date have been done in clinically defined cohorts of PD and other synucleinopathies, some with autopsy validation [164, 167, 168]. However, given the sensitivity of some of these assays in detecting aSyn seeds or clinicians may have to grapple shortly interpretation of a positive result in patients without a defined synucleinopathy syndrome, and it is not entirely clear if these patients are universally destined to phenoconvert. The integration of other biomarkers like hyposmia, polysomnograms for RBD, and DAT scans will likely further be of use to stratify those aSyn positive cases who are more likely to develop a parkinsonian syndrome. When combined with CSF or plasma biomarkers for AD, a more comprehensive picture of both primary and commonly occurring AD co-pathologies can be constructed for PD patients. These assays will likely prove useful in augmenting enrollment of homogenous populations into clinical trials. Focuses for future work to bring these skin immunofluorescence and aSyn-SAAs to clinical use include assay standardization and research in autopsy-confirmed cohorts to clarify the complex relationships between pathology in the brain and those detected from peripheral tissues and biofluids. aSyn assays that have quantitative value for disease activity remain a major unmet need, but the exciting development of these assays will allow for clinical assessments to be augmented by aSyn-specific biomarkers in a manner which has not been previously available for living patients.

## Supplementary Information

Below is the link to the electronic supplementary material.

Supplementary file1 (PDF 508 kb)

Supplementary file2 (PDF 4026 kb)
